# A Wearable Insole System to Measure Plantar Pressure and Shear for People with Diabetes

**DOI:** 10.3390/s23063126

**Published:** 2023-03-15

**Authors:** Jinghua Tang, Dan L. Bader, David Moser, Daniel J. Parker, Saeed Forghany, Christopher J. Nester, Liudi Jiang

**Affiliations:** 1 School of Engineering, University of Southampton, Southampton SO17 1BJ, UK; 2 School of Health Sciences, University of Southampton, Southampton SO17 1BJ, UK; 3 School of Health and Society, University of Salford, Salford M6 6PU, UK; 4 School of Allied Health Professions, Keele University, Keele, Newcastle ST5 5BG, UK

**Keywords:** diabetic foot ulcer, pressure, shear, insole system, plantar stress

## Abstract

Pressure coupled with shear stresses are the critical external factors for diabetic foot ulceration assessment and prevention. To date, a wearable system capable of measuring in-shoe multi-directional stresses for out-of-lab analysis has been elusive. The lack of an insole system capable of measuring plantar pressure and shear hinders the development of an effective foot ulcer prevention solution that could be potentially used in a daily living environment. This study reports the development of a first-of-its-kind sensorised insole system and its evaluation in laboratory settings and on human participants, indicating its potential as a wearable technology to be used in real-world applications. Laboratory evaluation revealed that the linearity error and accuracy error of the sensorised insole system were up to 3% and 5%, respectively. When evaluated on a healthy participant, change in footwear resulted in approximately 20%, 75% and 82% change in pressure, medial–lateral and anterior–posterior shear stress, respectively. When evaluated on diabetic participants, no notable difference in peak plantar pressure, as a result of wearing the sensorised insole, was measured. The preliminary results showed that the performance of the sensorised insole system is comparable to previously reported research devices. The system has adequate sensitivity to assist footwear assessment relevant to foot ulcer prevention and is safe to use for people with diabetes. The reported insole system presents the potential to help assess diabetic foot ulceration risk in a daily living environment underpinned by wearable pressure and shear sensing technologies.

## 1. Introduction

Approximately one in three people with diabetes develop a diabetic foot ulcer (DFU), and among them, one in four of them will progress to lower limb amputation [1,2]. The management of DFU is challenging as the risk of re-ulceration is 40% within the first year and 65% over five years [1]. The five-year survival rate after diabetes-related amputation is up to 50%, which is worse than breast and prostate cancers [3]. This evidence suggests that the current DFU prevention strategy, involving education, screening and foot care, in the UK National Health Service (NHS) is not fully effective and remains elusive. It is also well-recognised that a research-led solution is one of the key solutions to help address this issue [1,4,5]. Wearable devices adopting a user-centered design and using IoT technologies to monitor health conditions may offer a way to improve outcomes [6].

The development of DFU is a complex process, especially for people with combinations of peripheral neuropathy, peripheral arterial disease, and foot deformity. Neuropathy results in the loss of protective sensation, which in combination with a foot deformity or insufficient blood flow, leads to localised tissue injury and tissue death [7]. The load acting upon the foot includes pressure acting perpendicular and shear acting parallel to the surface of plantar tissue. Pressure is known to be one of the key external causes of DFU, and a threshold of 200 kPa has been advised as a target for pressure-relieving footwear and orthotic interventions for those who have previously ulcerated (measured under clinical conditions) [8]. Long-term and daily monitoring of pressure and providing alerts to patients when excessive pressure is identified have been shown to reduce ulceration risk [9]. However, The National Pressure Ulcer Advisory Panel et al. [10] reported that the combination of pressure and shear is responsible for ulceration. Bader et al. [11] reported that both pressure and shear exerted on the skin could cause internal shear stresses in the underlying tissues, which act to distort tissues, pinch and occlude capillaries crossing tissue planes, reduce blood and lymphatic flow and cause physical disruption of tissues and contribute to diabetic foot ulceration. Plantar tissue for people with diabetes also tends to have a reduced tolerance to external loading and, when coupled with bony prominences such as heel, metatarsal heads and hallux, further exacerbates ulceration risk. The IWGDF [12] has also long recognised that pressure is coupled with shear stress, and both have an impact on cell and tissue integrity. Both shear and pressure are therefore important for DFU risk assessment, and indeed, elevated shear stress has been reported at key sites at risk of plantar ulceration during walking under controlled laboratory conditions [13] but never in real-world conditions.

Insole systems that are sensitive to pressure but not shear have previously been developed for laboratory research purposes [14,15,16] as well as for the purpose of monitoring foot pressure in real-world living conditions. This includes the F-Scan System (Tekscan, Inc., Norwood, MA, USA), pedar (novel GmbH, München, Germany), XSENSOR (XSENSOR^®^ Technology Corporation, Calgary, AB, Canada) and Orpyx SI (Orpyx Medical Technologies Inc., Calgary, AB, Canada). However, none of these can measure shear forces at the same time when pressure is measured. To provide comprehensive assessment of plantar loading, tools were reported to measure multi-directional plantar forces but only in laboratory settings [13,17,18]. These include a strain gauge-based pressure and shear sensing platform which was designed only for barefoot conditions [13] and thus is not a wearable solution. Wang et al. [17] developed an inductive-based insole sensing system, which requires specific footwear modification and strapping electronic devices on the shank, limiting its adaptation to common footwear. Takano et al. [19] developed a system consisting of a combined shear force sensor and F-Scan pressure sensor; however, it requires a specialised insole, an electronic box to be worn and a wired connection to a computer, which again is not wearable in everyday living. Amemiya et al. [18] directly attached piezoelectric-based sensors to the metatarsal heads, and it is not a wearable system that could be worn by patients outside the lab. The motivation of this study is to develop a sensorised insole system that is capable of measuring both pressure and shear stress but also can be adapted to a range of footwear without modification. Such a wearable system could underpin a diabetic foot ulcer prevention solution based on comprehensive plantar pressure and shear monitoring during daily living activities. Based on a previously reported tri-axial pressure and shear (TRIPS) sensing system [20], a sensorised insole system capable of measuring both pressure and shear simultaneously has been developed. The TRIPS sensors are thin and flexible and have previously been applied at the residuum/socket interface of lower limb amputees to measure real-time kinetic residuum and socket interactions [20,21]. In this work, we focus on reporting the design, development and evaluation of the sensorised insole system which incorporates TRIPS sensing technology. The insole with sensor integration was evaluated using both laboratory-based and human participants tests. The potential of using this wearable insole system for future DFU prevention is discussed.

## 2. Development of the Sensorised Insole System

The TRIPS sensors’ working mechanism, design and development have been detailed in our previous publications [22]. In brief, a capacitive sensing mechanism was adopted to measure pressure and shear stresses (in two orthogonal directions) simultaneously as a function of time. Each sensor had an approximate dimension of 20 mm by 20 mm by 1 mm and was flexible. In this work, we focus on reporting the novel development of the sensorised insole system, which integrates these sensors ready for measuring pressure and shear across different plantar sites in real-time. Building upon a previously reported [20] single-sensor system, a bespoke electronic system was designed to incorporate multiple sensors, which requires additional power management, data storage and a system status indication module with a view to improving its usability in the daily living environment.

### 2.1. Sensor Locations

**Figure 1:**
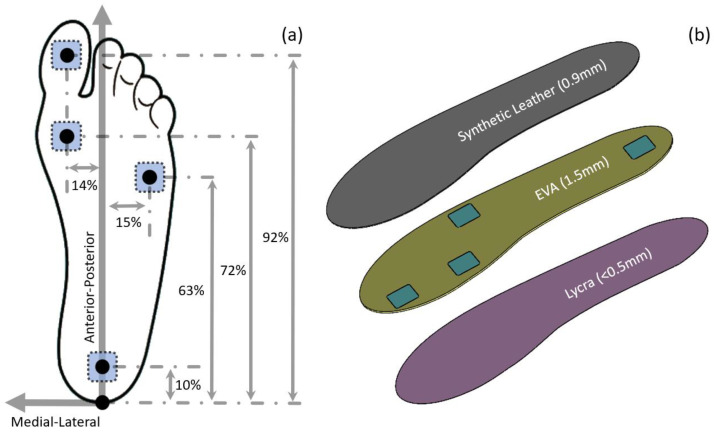
(**a**) Location of the sensors as a percentage of foot length and width. (**b**) Layered sensorised insole construction. The black dots represent the geometrical centre of the sensors.

The sensorised insole contains four TRIPS sensors, with the same dimensions (20 mm × 20 mm × 1 mm) and design, positioned at the heel, 5th metatarsal head (5MH), 1st metatarsal head (1MH) and hallux (Figure 1a). These locations were chosen as they represent the locations of the high occurrence of DFU and enable key gait events to be detected, for example, start and end of stance, heel-only and forefoot-only loading periods [23].

In the anterior–posterior direction, heel, 5MH, 1MH and hallux sensors were located at approximately 10%, 63%, 72% and 92% of the foot length measured from the posterior-most point. These percentages, in the anterior–posterior direction, were determined based on a foot morphological study [24] and a plantar pressure study [25]. In the medial–lateral direction of the heel, 5MH, 1MH and hallux sensors were located at approximately 0%, 15%, 14% and 15% of the foot width, measured from the long axis of the foot. These percentages, in the medial–lateral direction, were determined using plantar pressure distribution reported in previous studies [26,27].

### 2.2. Insole Construction

The sensorised insole (Figure 1b) consists of three layers of material, i.e., Ethylene-vinyl acetate or EVA (nora^®^ Lunacell, nora systems GmbH, Weinheim, Germany), synthetic leather (Yampi, A. Algeo Ltd., Liverpool, UK) and Lycra. These are the typical materials used for constructing a layered orthotic insole, as they demonstrate suitability for appropriate biocompatibility, durability and shock absorption against industry standards [28,29]. Sensors were embedded in the middle EVA layer. Four square cut-outs were made to the middle layer such that the sensor could be placed at the corresponding anatomical locations without protrusion. Subsequently, a layer of synthetic leather and a layer of Lycra material were adhered to the top and bottom surfaces of the middle layer, respectively. This was to ensure there no direct contact between the skin and the sensor to avoid elevated stress introduced by the sensors. The overall thickness of the insole was less than 3 mm and, therefore, could be used as a standalone insole or adhered to a prescribed insole to ensure its wider clinical application.

**Figure 2:**
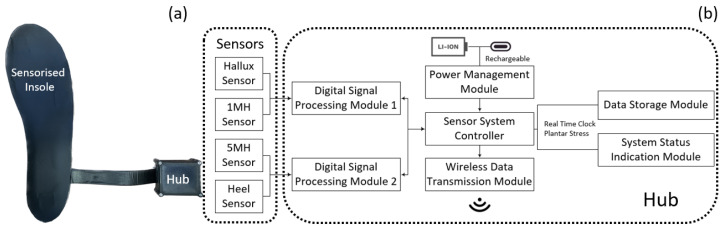
(**a**) A photo of the sensorised insole system and (**b**) a diagram illustrating key function modules within the hub.

The sensorised insole was connected to a signal processing and data collection hub via a thin and flexible cable, exiting from the posterior–lateral side of the insole, as shown in Figure 2a. The posterior–lateral exit was chosen for the flexible cable to avoid contact at the navicular region where the tissue is prone to injury. The hub can be attached to the lateral collar of the footwear with no modification required on users’ footwear to ensure the device is wearable in a daily living environment, which is critical for monitoring the risk of DFU.

### 2.3. Sensorised Insole System

Figure 2b illustrates the functional diagrams of the electronic system within the hub, formed by key sub-modules. The sensorised insole system consists of a sensorised insole and a hub containing an electronic system for data acquisition and processing. Four sensors were incorporated within an insole, forming a sensorised insole. The operating mechanism of the hub is detailed in a previous publication [20]. In brief, the main functionalities of the electronic hub system are controlled by a 32-bit microcontroller loaded with a real-time operating system which runs multi-threaded applications to manage tasks for each module, as shown in Figure 2b. Signals from the sensorised insole are processed by the digital signal processing module, containing capacitance-to-digital converters, at 100 Hz operating frequency. The digitised sensor signals are then communicated with the sensor system controller via the serial–peripheral interface. The sensor system controller subsequently sends both plantar stress data and real-time clock data to an onboard data storage module via the secure-digital input–output interface for data storage purposes. This provides the capability that plantar stress can be studied as a function of real-time in a year–month–day–hour–minutes format. The hub also provides a wireless data transfer function, so the data can be communicated wirelessly with an external device, such as a mobile phone. From a user perspective, a USB type-C connector is available on the hub for charging purposes, and a simple LED light, controlled by the system status indication module, is provided to the user for hub system status indication.

## 3. Laboratory Evaluation of the Sensorised Insole System

### 3.1. Experimental Setup and Test Method

**Figure 3:**
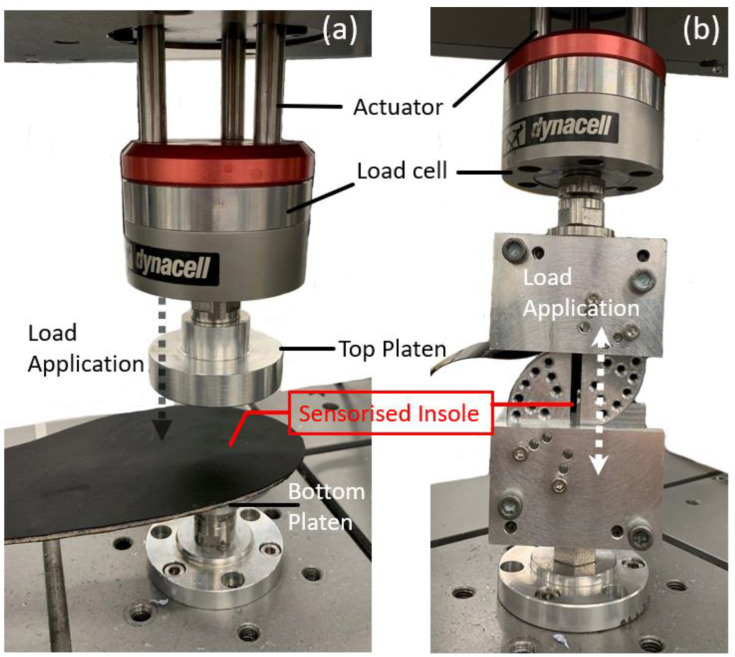
Experimental setup for evaluating (**a**) pressure and (**b**) shear stress measurement from the insole system.

A uniaxial mechanical test machine (E1000, Instron, High Wycombe, UK) with a load cell capacity of ±1 kN was used to evaluate the performance of the insole system. Aluminium platens were designed, manufactured and attached to the test machine with a view of applying known pressure (Figure 3a) and shear stresses (Figure 3b) to the specified sensor location of the sensorised insole. Static and dynamic loading profiles were designed, and the test machine was programmed to convert the design loading profile to actuator movements. The known applied load from the test machine was then compared with the outputs of our sensorised insole system.

### 3.2. Pressure

**Figure 4:**
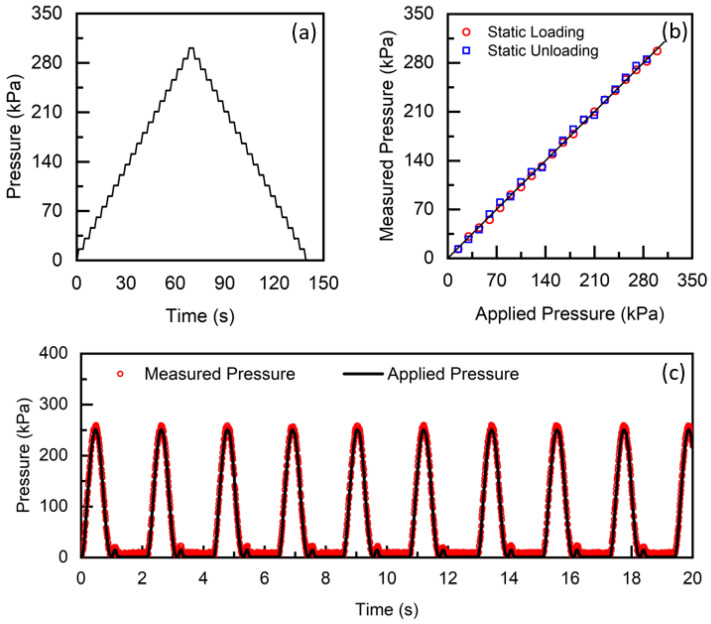
(**a**) Applied static pressure from the Instron mechanical test machine as a function of time. Measured pressure from the insole system and applied pressure from the test machine, obtained from the (**b**) static and (**c**) dynamic pressure test.

A step loading profile (Figure 4a), incorporating 20 loading and unloading steps with 10 kPa pressure per step, was designed to characterise static pressure measurement from the insole system. In static conditions, a linearity error of 2% was estimated in a measurement range between 0 kPa and 300 kPa (Figure 4b). The cyclic loading profile was designed to evaluate the insole system performance in a controlled laboratory environment by applying representative load experienced during walking. The profile consists of a half sinusoidal wave with a loading amplitude of 250 kPa and a frequency of 1 Hz, followed by an unloading period of approximately 0.5 s. Accuracy error, the estimated percentage of the peak value, is approximately 4% of the full scale in both static and dynamic test conditions.

### 3.3. Shear Stress

**Figure 5:**
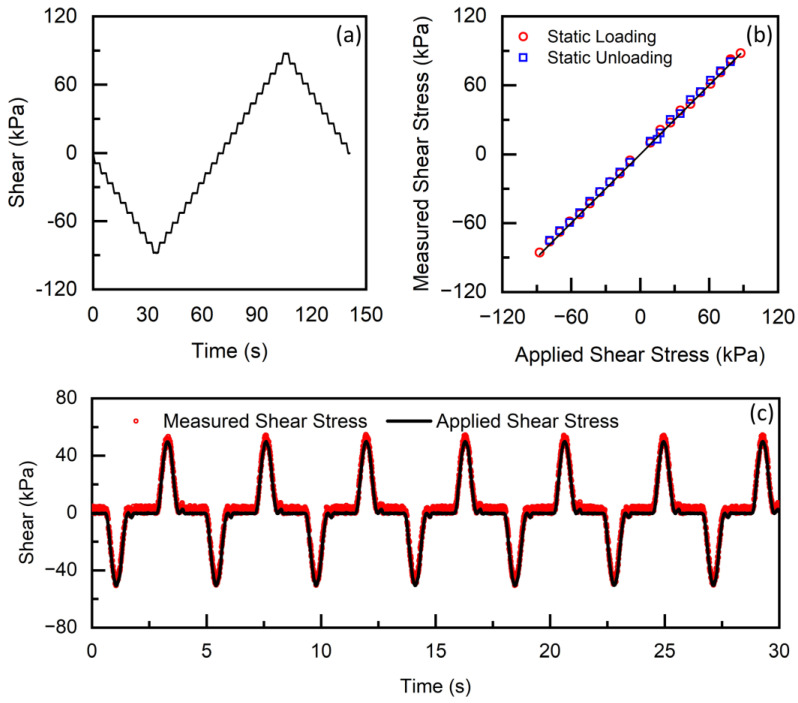
(**a**) Applied static shear stress from the mechanical test machine as a function of time. Measured shear stress from the insole system and applied shear stress from the test machine, obtained from the (**b**) static and (**c**) dynamic shear test.

Similar step-loading profiles were designed to evaluate shear stress measurement from the insole system in a static condition. The step profile consists of 10 loading and unloading steps in both positive and negative directions (Figure 5a). Each loading step corresponds to 9 kPa of shear stress increment. In static conditions, a linearity error of up to 3% was estimated in a measurement range between −90 kPa and 90 kPa. A dynamic shear stress profile was designed such that a half-sinusoidal loading profile was applied with an amplitude of 50 kPa in both positive and negative directions at 1 Hz loading frequency. Followed by the dynamic load phase, an unloading phase of up to 0.5 s was also incorporated. In dynamic conditions, the accuracy error is estimated to be 5% of the full scale.

Stress measurements from the insole system were evaluated in this study. Low linearity errors of up to 3% were revealed in both pressure and shear measurement. The accuracy error (up to 5% of full scale in both pressure and shear) of the insole system reported in this study is equivalent to a recently reported SLIPS system [17], as well as a commercial pressure-only system [30].

## 4. Evaluation of the Sensorised Insole System on a Human Participant

### 4.1. Test Protocol

**Figure 6:**
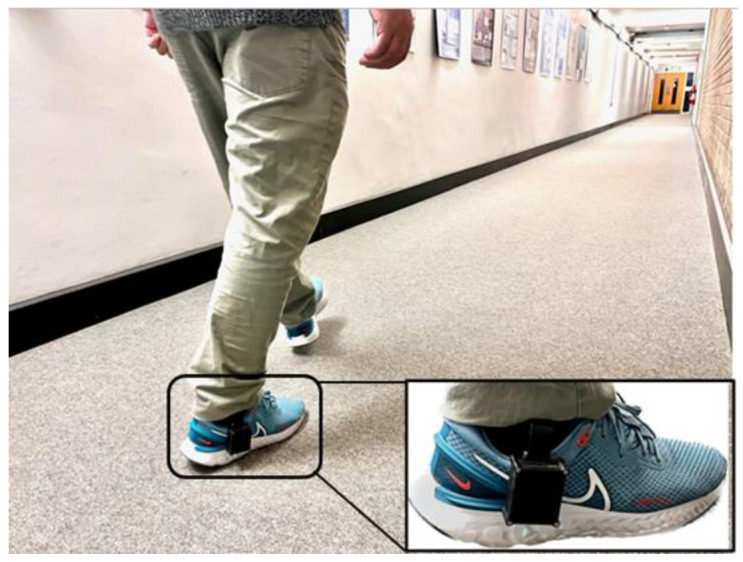
A photo showing level walking along a 28 m indoor corridor with the device attached to the footwear.

One healthy male participant (age 32 years, body mass 97 kg, height 177 cm, UK shoe size 8) with no lower limb injury, or known walking dysfunctions, was recruited for walking tests. The participant was asked to change into a pair of standard socks and trainers (React Miler 3, Nike Inc., Beaverton, OR, USA). The original insole in the trainer was removed and replaced with the sensorised insole. The participant walked for at least five minutes to ensure comfort at the start. Subsequently, he was asked to perform level walking along a 28 m corridor (Figure 6) at a self-selected speed. Walking cadence was recorded by counting the number of steps covered in 30 s and used to define self-selected walking cadence.

**Figure 7:**
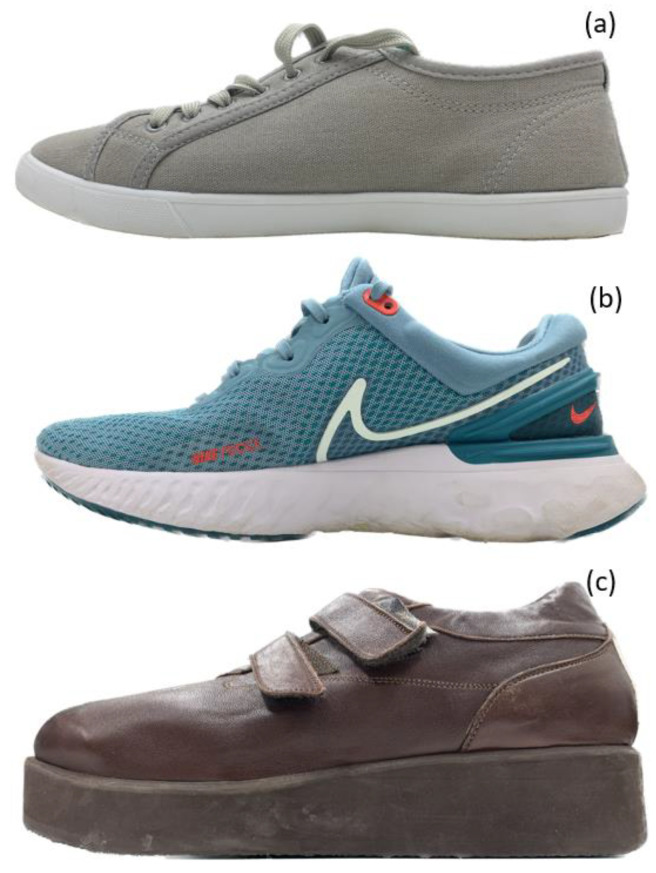
(**a**) Plimsoll with a flat sole, (**b**) trainer as a standard type of footwear used in the experiment and (**c**) therapeutic footwear with rocker features.

The level walking test was repeated with two additional types of footwear (Figure 7). Plimsolls (Figure 7a) and therapeutic footwear (Figure 7c). The plimsoll has a flat outsole, representing typical retail footwear that would not be advised for people with diabetes due to the lack of sole thickness and inadequate upper support. The therapeutic footwear (Omar 11, fisio duna) was designed for people with diabetes [31] and had a forefoot rocker angle of 20°. The self-selected walking cadence was controlled by a digital metronome to minimise the effect of walking speed on plantar pressure and shear measurement.

### 4.2. Temporal Pressure and Shear Stress Profile during Level Walking

**Figure 8:**
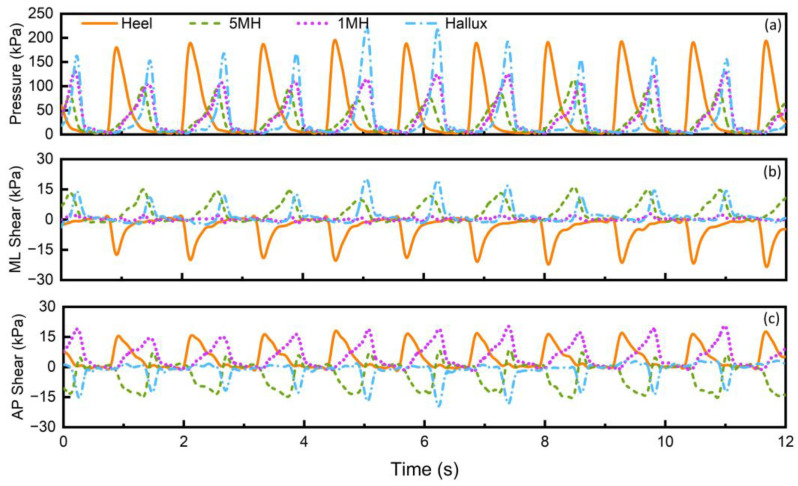
(**a**) Pressure, (**b**) medial–lateral (ML) shear and (**c**) anterior–posterior (AP) shear stress as a function of time from the healthy participant wearing a trainer.

Figure 8 shows the typical pressure, medial–lateral and anterior–posterior shear stress obtained from a healthy participant as a function of time when wearing a pair of everyday trainers. Peak pressure of up to 200 kPa was obtained across the four locations (Figure 8a). Within the stance phase, four distinctive peaks were revealed, with peak pressure at the heel revealed first in the initial contact phase of the gait and peak pressure at the hallux revealed at last at the hallux location, representing the push-off phase of the gait. These sequence-related peak events, as well as the timing between each of the two peaks, could be metrics of the roll-over characteristics of the foot, important as people with diabetes can experience loss of ankle range of motion and impaired gait as a result [32]. It is also important to note that in-shoe pressure of 200 kPa has been previously recommended by IWGDF as an indicative threshold to help prevent recurrent foot ulceration risk for people with diabetes. The real-time pressure and corresponding plantar sites reported here could also be potentially explored to facilitate the assessment.

Figure 8b,c illustrates the shear stress in the medial–lateral direction and anterior–posterior direction, respectively. Up to 18 kPa and 16 kPa of peak shear stress were measured in the medial–lateral and anterior–posterior directions across the four locations, respectively. The peak shear stress reported in this study is lower than that measured barefoot, highlighting the difference between in-shoe and barefoot results [33]. It is also worth noting that the peak shear stress was significantly lower than peak pressure, which is consistent with previous studies [13,17]. To our best knowledge, this is the first study that reports in-shoe real-time shear stress in two orthogonal directions, which could be potentially used to study balance in the medial–lateral direction as well as braking and propulsive impulses during gait [34]. These are critical parameters as understanding balance may help better manage the risks of loading asymmetry due to loss of movement control and localised stress distributions, all of which may lead to ulceration [35].

### 4.3. Effect of Footwear on Plantar Pressure and Shear Stresses

**Figure 9:**
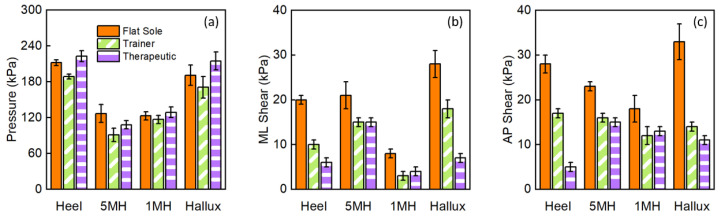
(**a**) Mean peak pressure and (**b**) medial–lateral (ML) and (**c**) anterior–posterior (AP) shear stress obtained over gait cycles with three types of footwear.

Figure 9a illustrates the mean peak pressure (MPP) obtained at the four locations when wearing three types of footwear. Regardless of the footwear, higher pressures were obtained at the heel (up to 215 kPa) and hallux (up to 243 kPa) compared to the other two metatarsal locations. At all locations, the lowest pressures were obtained when wearing trainers compared to the value obtained with therapeutic and flat-sole footwear. The reduction in peak pressure of up to 20% in all four locations, when wearing trainers may be attributed to the mechanical property, e.g., Young’s Modulus, as well as the microstructure of the material used for the footwear construction to achieve shock absorptions. The plimsoll and therapeutic footwear featured thin and rigid outsoles, respectively, which may have reduced the shock absorption capability.

Among the four locations, the highest shear stress of up to 28 kPa and 33 kPa was revealed at the hallux location when wearing plimsolls, in medial–lateral and anterior–posterior directions, respectively. At all four locations, reductions of up to 75% medial–lateral shear and 82% anterior–posterior shear were evident when wearing therapeutic footwear compared to the plimsolls. This may be explained by the rocker sole (Figure 7c) incorporated in the therapeutic footwear design. In the early stance phase, the heel rocker assists the foot lowering to achieve foot flat in the midstance phase. In the terminal stance phase, the forefoot rocker helps transfer the load from the hindfoot to the forefoot and thereby achieve foot ‘roll-over’. Both these footwear features were absent in the plimsolls, requiring the activation of muscle forces to assist load transfer under the foot, generating different shear stresses at the plantar interface. In addition, up to 40% and 61% reduction in medial–lateral shear was revealed when wearing the therapeutic footwear compared to that obtained for the trainer at the heel and hallux, respectively. Similar shear stress reduction was also revealed in the anterior–posterior direction, where reductions of up to 71% and 21% were measured at the heel and hallux, respectively. This indicates that the reported insole system has adequate sensitivity and could detect expected differences in the effects of the trainer and therapeutic footwear, which have similar footwear construction features.

The combined pressure and shear assessment may be used to offer insights to understand the effect of the design of footwear on loading characteristics at critical anatomical locations. This preliminary case study shows that pressure alone is not adequate to provide a comprehensive assessment of loading characteristics as a function of footwear design and choice. The significant difference in shear stress revealed when wearing therapeutic footwear may be potentially used as quantitative evidence to assist the design of footwear for DFU prevention.

## 5. Safety Evaluation for Use in Shoes by Patients with Diabetes

### 5.1. Test Protocol

Five participants, including three males and two females with diabetes at risk of ulceration, were recruited to participate in a walking evaluation. The primary aim was to detect whether the usage of the sensorised insole would induce notable changes in pressure for people with diabetes. Participants had a mean age of 67.2 years (range: 40–85 years) and UK shoe size between 8 and 9 with known diabetes duration 10.8 years (range: 2–22 years). The risk of foot ulceration was assessed on all participants based on IWGDF guidelines, resulting in four participants with moderate and one with a high risk of DFU. Participants completed walking at a self-selected pace along a 50 m walkway whilst wearing standardised therapeutic footwear (Omar 11, fisio duna) with and without the sensorised insole.

**Figure 10:**
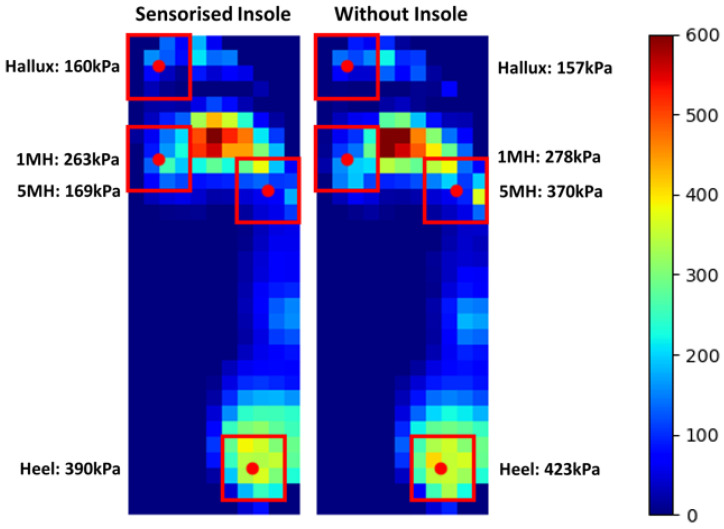
Mean peak plantar pressure distribution during walking obtained using the XSENSOR system with and without the sensorised insole. The four sensing locations are highlighted to allow regional peak pressure value comparison. The red dots represent the geometrical centre of each sensor.

Plantar pressure data were collected using the XSENSOR system (Foot and Gait v4, XSENSOR^®^ Technology Corporation, Calgary, AB, Canada) at 50 Hz. To evaluate the safety of wearing the new insole system, the difference in MPP over 10 mid-gait steps was calculated [36] (Table 1); this represents a known marker for risk in the diabetic foot [12]. This was evaluated for regions of interest defined based on sensor locations stated in Figure 1a, with an additional boundary of 10% in each direction to accommodate for misalignment (Figure 10). The group mean differences were then calculated.

### 5.2. Safety Evaluation on People with Diabetes

Figure 10 illustrates the comparison of regions of interest for the peak pressure distribution map with and without the sensorised insole. Table 1 presents the MPP outcomes for each participant. The incorporation of the sensor within the insole resulted in −9%, −41%, −16% and −11% group mean percentage difference in peak pressure during walking at the heel, 5MH, 1MH and hallux, respectively. The 5MH region may also be affected by the raised lateral border of the XSENSOR measurement insole [30]. Due to the slight padding of the sensorised insole’s middle EVA layer, some reduction in pressure was observed across regions. The effect within individuals and at individual regions varied, with changes in pressure affected by proximity to other loaded sites and variation within the gait. The use of small and fixed pressure masking associated with sensor locations may have influenced the step-to-step variability. For sites which demonstrated increased pressure, the resulting change in pressure magnitude was less than or similar to the between-step standard deviation suggesting this may be underpinned by step-to-step variation. These changes are, therefore, beneficial or negligible and show that the sensorised insole introduced almost no risk to user comfort and tissue injury.

## 6. Discussion

This paper presents an insole system that can measure real-time pressure and shear stresses under the foot. The design included all the elements required for a practical at-home solution, including a data storage interface, battery charging and mounting to footwear. The system is suitable for the assessment of the complex loading characteristics of people with diabetes and may inform guidance and management to underpin DFU prevention. In addition, the two-directional shear stresses, coupled with pressure, can be exploited to study balance in both sagittal and coronal planes, braking and propulsive impulses in people with diabetes and others affected by difficulties of movement control. Further work should seek to understand these kinetic parameters coupled with lower limb kinematics to provide a comprehensive biomechanical assessment of the foot in real-world settings of people’s daily lives and activities.

The sensorised insole can be used in footwear with no modification or customisation required, assuming suitable footwear is chosen. This supports its use in daily living environments as a monitoring tool to provide warning to patients and health professionals when pressure and shear-related elevated DFU risks are detected. The insole presented in this study offers a significant advantage compared to other devices [17,18], where footwear modification is required, or over-sized device electronics are required to be attached to other parts of the lower limb, which may affect normal walking and also impact adherence and usage. These factors were subjected to further study as part of this project.

The footwear used in this study represents the range of footwear available, including those offered for patients who have diabetes and are classified as at-risk of ulceration [37]. While therapeutic footwear is the recommended footwear for patients at high risk of ulceration [12], this is not standard provision across patients of lower risk. So, understanding the use of the insole system in a range of footwear and what changes to pressure and shear might occur due to different footwear is an important next step in research. Pressure values do not demonstrate large changes even across this known range of footwear; however, shear data presented in Figure 9 show potential for modification by footwear intervention and warrants further investigation.

While initial work has highlighted the importance of activity type in plantar pressure assessment [38], it is unknown how these varied activities of daily living generate potential risk from shear loading for people with diabetes. Further, the sensorised insole presented here will enable measurements relevant to individual patients’ activity profiles, allowing for a more personalised monitoring and risk evaluation in a real-world setting. To facilitate these future studies, further work in assessing the performance of the sensorised insole in real-world conditions such as weather, different ground surfaces and terrains will be conducted.

## 7. Conclusions

A first-of-its-kind sensorised insole system was reported, which is capable of measuring real-time plantar pressure and shear stress that could be potentially used by people with diabetes to help monitor and assess the risk of DFU. The technical performance of the system was validated through a combination of lab testing and initial walking trials. The insole and the wireless electronic hub were designed to be used with a range of existing footwear without the need for modifications. This is a significant improvement over any other existing devices reported in this field. These important wearability features and the comprehensive in-shoe pressure and shear measurement capability are essential for DFU prevention in the daily living environment. Preliminary results involving a healthy participant revealed such a wearable system is also sensitive to investigating the effect of different footwear on plantar loading. The safety of the device was further evaluated in diabetic participants. The result suggests that the inclusion of the sensorised insole itself does not elevate the plantar pressure and thus introduces no risk to user comfort and plantar tissue injury. Overall, our initial results reported here demonstrated the significant potential for the use of the sensorised insole in everyday living for DFU risk monitoring and prevention.

## 8. Future Work

Future work should involve recruiting people with diabetes with different levels of DFU risks to investigate the association between the plantar loading profile and the formation of DFU. Data from one participant (UK shoe size 8) were reported here to underpin the technological development and potential suitability for people with diabetes. Sensorised insoles of different sizes should be designed to accommodate the need of an expanded population, and subsequently, device durability tests must be conducted. The potential acceptance of the device by a large population would also help drive the unit cost down.

## Figures and Tables

**Table 1:** Peak pressure safety evaluation for 5 participants with diabetes. MPP: Mean peak pressure values for each participant represent the average of 10 mid-gait steps. Effect calculated as absolute pressure with sensorised insole MPP minus without insole MPP (S − W).

	Sensorised Insole	Without Insole	Effect		
D_01	Mean ± SD	Mean ± SD	S − W	% Diff	
Heel	119.46 ± 10.98	118.90 ± 11.57	0.57	0%	-
5MH	46.83 ± 3.30	31.58 ± 4.06	15.25	33%	/\
1MH	74.60 ± 3.88	85.68 ± 10.43	−11.08	−15%	\/
Hallux	171.45 ± 28.71	208.02 ± 15.54	−36.57	−21%	\/
D_02	Mean ± SD	Mean ± SD	S − W	% Diff	
Heel	178.25 ± 20.56	211.37 ± 16.04	−33.12	−19%	\/
5MH	92.84 ± 14.69	154.44 ± 34.51	−61.59	−66%	\/
1MH	284.38 ± 28.62	308.89 ± 61.47	−24.51	−9%	\/
Hallux	123.94 ± 20.11	172.68 ± 26.08	−48.74	−39%	\/
D_03	Mean ± SD	Mean ± SD	S − W	% Diff	
Heel	197.75 ± 26.18	185.24 ± 19.99	12.51	6%	/\
5MH	94.45 ± 19.25	82.94 ± 10.74	11.51	12%	/\
1MH	187.31 ± 53.43	257.36 ± 42.90	−70.05	−37%	\/
Hallux	244.82 ± 15.83	253.46 ± 27.35	−8.65	−4%	\/
D_04	Mean ± SD	Mean ± SD	S − W	% Diff	
Heel	389.68 ± 19.89	422.73 ± 20.10	−33.05	−8%	\/
5MH	168.99 ± 28.70	370.31 ± 62.10	−201.32	−119%	\/
1MH	262.58 ± 53.02	277.80 ± 11.28	−15.22	−6%	\/
Hallux	159.82 ± 14.16	156.85 ± 7.61	2.97	2%	/\
D_05	Mean ± SD	Mean ± SD	S − W	% Diff	
Heel	319.48 ± 9.26	397.56 ± 33.17	−78.07	−24%	\/
5MH	168.76 ± 14.50	273.22 ± 41.83	−104.46	−62%	\/
1MH	333.30 ± 53.14	381.77 ± 46.23	−48.46	−15%	\/
Hallux	304.76 ± 49.74	277.67 ± 39.40	27.09	9%	/\

## Data Availability

All data supporting this study are openly available from the University of Southampton repository at https://doi.org/10.5258/SOTON/D2560 (accessed on 19 February 2023).
